# “What is the actual goal of the pathway?”: examining emergency department physician and nurse perspectives on the implementation of a pediatric concussion pathway using the theoretical domains framework

**DOI:** 10.1186/s12913-021-06110-2

**Published:** 2021-02-05

**Authors:** Anh Ly, Roger Zemek, Bruce Wright, Jennifer Zwicker, Kathryn Schneider, Angelo Mikrogianakis, Alf Conradi, David Johnson, Brenda Clark, Karen Barlow, Joseph Burey, Ash Kolstad, Keith Owen Yeates

**Affiliations:** 1 Department of Psychology, University of Calgary, 2500 University Drive NW, Calgary, Alberta T2N 1N4 Canada; 2 Department of Pediatrics, University of Ottawa, 75 Laurier Avenue East, Ottawa, Ontario K1N 6N5 Canada; 3 Department of Pediatrics, University of Alberta, 3-513 Edmonton Clinic Health Academy, Edmonton, Alberta T6G 2R7 Canada; 4 University of Calgary, School of Public Policy, 906 8th Avenue SW, Calgary, Alberta T2P 1H9 Canada; 5 University of Calgary, Faculty of Kinesiology, 2500 University Drive NW, Calgary, Alberta T2N 1N4 Canada; 6 Department of Pediatrics, McMaster University, 1280 Main Street, Hamilton, Ontario L8S 4K1 Canada; 7 Department of Pediatrics, University of Alberta, 4-539 Edmonton Clinic Health Academy, Edmonton, Alberta T6G 2R7 Canada; 8 Department of Pediatrics, University of Calgary, 28 Oki Drive NW, Calgary, T3B 6A8 Canada; 9 Department of Pediatrics, University of Alberta, 10230 111 Avenue, Edmonton, Alberta T5G 0B7 Canada; 10 University of Queensland, Child Health Research Centre, Brisbane, QLD 4072 Australia; 11 Department of Psychology, University of Windsor, 401 Sunset Avenue, Windsor, Ontario N9B 3P4 Canada

**Keywords:** Pediatric concussion, Clinical pathway, Implementation, Emergency care, Theoretical domains framework, Health outcomes, Standardization

## Abstract

**Background:**

Multiple evidence-based clinical practice guidelines (CPGs) exist to guide the management of concussion in children, but few have been translated into clinical pathways (CP), which operationalize guidelines into accessible and actionable algorithms that can be more readily implemented by health care providers. This study aimed to identify the clinical behaviours, attitudinal factors, and environmental contexts that potentially influence the implementation of a clinical pathway for pediatric concussion.

**Methods:**

Semi-structured interviews were conducted from October 2017 to January 2018 with 42 emergency department clinicians (17 physicians, 25 nurses) at five urban emergency departments in Alberta, Canada. A Theoretical Domains Framework (TDF)-informed interview guide contained open-ended questions intended to gather feedback on the proposed pathway developed for the study, as well as factors that could potentially influence its implementation.

**Results:**

The original 14 domains of the TDF were collapsed into 6 clusters based on significant overlap between domains in the issues discussed by clinicians: 1) knowledge, skills, and practice; 2) professional roles and identity; 3) attitudes, beliefs, and motivations; 4) goals and priorities; 5) local context and resources; and 6) engagement and collaboration. The 6 clusters identified in the interviews each reflect 2–4 predominant topics that can be condensed into six overarching themes regarding clinicians’ views on the implementation of a concussion CP: 1) standardization in the midst of evolving research; 2) clarifying and communicating goals; 3) knowledge dissemination and alignment of information; 4) a team-oriented approach; 5) site engagement; and 6) streamlining clinical processes.

**Conclusion:**

Application of a comprehensive, evidence-based, and theory-driven framework in conjunction with an inductive thematic analysis approach enabled six themes to emerge as to how to successfullly implement a concussion CP.

## Background

Pediatric concussion is a significant public health burden, sometimes referred to as a silent epidemic [1]. An estimated 1–2 million children in North America sustain concussions annually, with those seeking medical care rising dramatically [2, 3]. Children with concussion often report postconcussive symptoms, including somatic (e.g., headache, dizziness), cognitive (e.g., inattention, forgetfulness), and affective (e.g., irritability, dysphoria) complaints [4]. Postconcussive symptoms are most severe acutely, but can persist for weeks to months and result in functional disability and declines in quality of life in 15–25% of children [5–8]. Moreover, postconcussive symptoms often disrupt daily activities, with some children experiencing associated difficulties in social and academic settings [9, 10].

Multiple evidence-based clinical practice guidelines (CPGs) have been developed to guide the management of pediatric concussion [1, 11–13]. However, their implementation in clinical settings is inconsistent, thus perhaps explaining the observed significant practice variation and knowledge gaps. For instance, in studies of primary care providers and emergency physicians in Ontario, Canada who diagnose and manage concussion, only 47 and 60% recommended an initial period of school absence, 37 and 64% correctly applied return-to-play guidelines, and 26 and 22% reported regular use of standardized rating scales for assessing concussion, respectively [14, 15]. Variation in clinical practice can be associated with increased risk, including premature return to school and sport, which has been reported to occur in 44.7 and 43.5% of cases, respectively, for children with sport-related concussions [16].

Two major factors possibly account for the lack of knowledge translation in the clinical care of pediatric concussion. First, CPGs for pediatric concussion have seldom been translated into clinical pathways (CPs), which operationalize CPGs into accessible and actionable algorithms for provider use [17, 18]. Second, implementation typically relies on passive dissemination, rather than planned interventions. Effective interventions require evidence-based, theory-driven approaches to systematically evaluate and address factors that may affect uptake of CPs [19, 20].

Recently, the Theoretical Domains Framework (TDF) has shown promise as a theory-based approach for intervention planning [21]. The TDF provides a comprehensive framework of 14 theoretical domains [22], based on 33 behaviour change theories, that provide concrete guidance for assessing factors that may influence intervention implementation [23–26]. Hence, it serves as a useful approach to design interventions by anticipating relevant implementation challenges. To date, the TDF has been applied to a wide range of clinical settings and issues, from public health prevention planning to prescribing behaviour among providers [27–37].

Among its advantages, the TDF allows for implementation to be linked to underlying theories of behaviour change. The framework’s comprehensive coverage encompasses a broad scope of influences on behaviour, rather than a limited set represented by any particular theory. Hence, implementation challenges can be addressed directly based on the explicit linkage between *theories* and *techniques* of behaviour change [22–25], thereby promoting change in the clinical setting based on evidence-based principles.

In 2015, the Maternal Newborn Child Youth (MNCY) Strategic Clinical Network (SCN) of Alberta Health Services (AHS) identified pediatric concussion as one of its three top priorities, based in part on provincial data showing that the number of children diagnosed with concussion had doubled in the past 10 years, and convened a work group to develop best-practice, evidence-based CPs to guide the management of pediatric concussion in both emergency department (ED) and primary care settings. With support from AHS and the Brain Canada Foundation**,** the authors sought to conduct an expanded evaluation of the implementation and impact of the CP for acute care of pediatric concussion across five EDs in Alberta, Canada. The parent project involved a stepped-wedge cluster randomized trial to examine the health outcomes associated with a CP for the management of pediatric concussion in the ED. The decision to focus on the ED reflected provincial data showing that the large majority of diagnoses of concussion in Alberta occur in the ED.

Prior to implementing the CP and conducting the trial, we conducted qualitative interviews with ED clinicians to gather feedback on the proposed CP, as well as factors that could potentially influence its implementation. The primary aim of the current study was to analyze the interviews to identify the clinical behaviours, attitudinal factors, and environmental contexts that might potentially influence CP implementation. A secondary aim of the study was to assess the utility of the TDF as a qualitative research tool to achieve the primary aim.

## Methods

### Participants and recruitment

A total of 42 ED clinicians (17 physicians, 25 nurses) participated in semi-structured interviews to share their experiences in providing pediatric concussion care along with their views on the barriers and facilitators of implementing a CP. Clinicians were recruited through convenience and snowball sampling from 5 EDs in urban hospitals in Alberta. A recruitment notice was initially sent to all physician and nursing staff at participating research sites through key site leads (e.g., section chiefs, physician unit leads, nurse unit managers), for a total of approximately 800 nurses and 150 physicians. Those interested voluntarily contacted the study coordinator to arrange an interview. After interviewing a few participants, we used a snowball sampling strategy by asking these individuals to help put us in contact with nurse educators who might be interested in taking part in the study. This purposive sampling strategy was used to ensure that nurse educators from each site were represented in the final sample because they play a key role in EDs for disseminating information about new clinical initiatives to staff.

### Procedures

From October 2017 to January 2018, interviews were conducted in person by the study coordinator (AL). Interviews lasted approximately 45–60 min each and were audio-recorded, transcribed verbatim, and de-identified prior to analysis. Extensive field notes were taken during the interviews to capture contextual factors and nonverbal aspects of the interview. The notes were systematically summarized after each interview to capture immediate impressions and to record key statements or emerging themes to allow for development of a preliminary coding schema for further analysis. A semi-structured interview guide was developed using the TDF based on feedback from our multidisciplinary research team, which included a clinical neuropsychologist, emergency physicians, a neurologist, a developmental-behavioural pediatrician, a critical care physician, a physiotherapist, community representatives with a history of concussion, and concussion researchers. The interview guide included open-ended questions structured to reflect the 14 domains of the TDF (see Table 1).

**Table 1:** Semi-structured topic guide for clinician interviews

Domain	Sample Questions
1. Knowledge	To your knowledge, what are the existing guidelines for the diagnosis and management of concussion in children? Are there aspects of pediatric concussion that you would like to learn more about? What is the best way for you to learn about the clinical pathway?
2. Skills	How would you describe the training you’ve received in pediatric concussion care?
3. Social/professional role and identity	Given your role, which aspect of pediatric concussion care do you feel you are most responsible for? Would the introduction of a clinical pathway affect your role? How?
4. Beliefs about capabilities	How would you describe your level of confidence around being asked to implement a clinical pathway for concussion?
5. Optimism	Do you think that the implementation of a clinical pathway will make any difference? Do you anticipate that there will be any barriers to implementing the clinical pathway?
6. Beliefs about consequences	In your opinion, what do you think are the possible negative aspects of implementing a clinical pathway?
7. Reinforcement	What are the best ways to ensure that clinical staff will use the clinical pathway as expected?
8. Intentions	Currently, how motivated would you say you are to learn about and implement a new clinical pathway in your practice? Do you anticipate this level of motivation could change over time? If so, why?
9. Goals	Given your clinical responsibilities, how would you prioritize the clinical pathway implementation relative to other activities?
10. Memory, attention, and decision	What are some ways we can support you, other clinicians, and the clinic in general in implementing a clinical pathway?
11. Environmental context	What resources are available to support you or the clinic in general in the implementation of a clinical pathway? Can you think of any organizational limitations that you feel would hinder your ability to implement the clinical pathway effectively?
12. Social influences	Are there factors such as interpersonal relations among colleagues or professional roles and boundaries that could impact the implementation of a clinical pathway?
13. Emotion	Do you foresee any potential for the clinical pathway implementation to elicit negative emotions among clinicians or hospital administrators?
14. Behavioral regulation	In terms of your personal practice, what are the mechanisms that will help you ensure that you regularly and effectively implement the clinical pathway?

The study was approved by the Health Research Ethics Boards of both the University of Calgary and University of Alberta (REB17–1543). Signed informed consent was obtained from all participants prior to interviews.

### Analysis

An inductive thematic analysis was conducted following the analytic approach proposed by Bengtsson [38]. Transcribed interviews were analyzed using NVivo to identify common codes, sub-headings, generic categories, and emergent themes. Interviews with physicians and nurses were analyzed together. The lead author (AL) conducted the primary analysis and independently coded the transcripts. Preliminary categories and emergent themes were then discussed with the principal investigator (KOY), who provided critical feedback. In the second-level analysis, the lead author re-examined the transcripts to collapse some categories, identify patterns in the data, and synthesize findings to determine the final themes that corresponded to each domain of the TDF. The final synthesis and interpretation involved further discussions with the principal investigator, who reviewed a detailed summary analysis containing descriptions of themes, supportive evidence from the interviews, and a discussion of the implications of the findings for the CP implementation. The interview summary was shared with the larger research team to garner feedback and to discuss practical steps for the next phase of the study, which involved working with clinical sites to develop site-specific implementation strategies. Predominant themes were those that participants mentioned most frequently or discussed extensively in relation to considerations for implementing a CP, and were deemed by the investigators to offer valuable insight into guiding the project’s goals.

## Results

A total of 17 ED physicians and 25 nurses participated in the interviews. Of the physicians, all of whom had specialized training in emergency medicine, 8 had additional specialized training in pediatrics and 4 in sports medicine. Nurse participants included 9 general nurses, 4 unit managers, 8 nurse educators, 2 licensed practical nurses, and 2 in specialized administrative roles. Overall, the participants were diverse in terms of personal and practice characteristics, including sex, years of practice, clinical role, and experience in pediatric care (see Table 2).

**Table 2:** Characteristics of emergency clinicians interviewed

	MDs	RNs	Total
Calgary^a^			
Site 1 (Academic Hospital)	4	5	9
Site 2 (Satellite Hospital)	–	5	5
Edmonton			
Site 3 (Academic Hospital)	5	4	9
Site 4 (Community Hospital)	3	5	8
Site 5 (Community Health Centre)	5	6	11
Sex			
Male	9	1	10 (23.8%)
Female	8	24	32 (76.2%)
Years of Practice			
< 5 years	4 (23.5%)	7 (28.0%)	11 (26.2%)
5–10 years	5 (29.4%)	7 (28.0%)	12 (28.6%)
11–20 years	5 (29.4%)	3 (12.0%)	8 (19.0%)
≥ 20 years	3 (17.6%)	8 (32.0%)	11 (26.2%)

^a^the same physician group provides service at Site 1 and Site 2 in Calgary

**Fig. 1:**
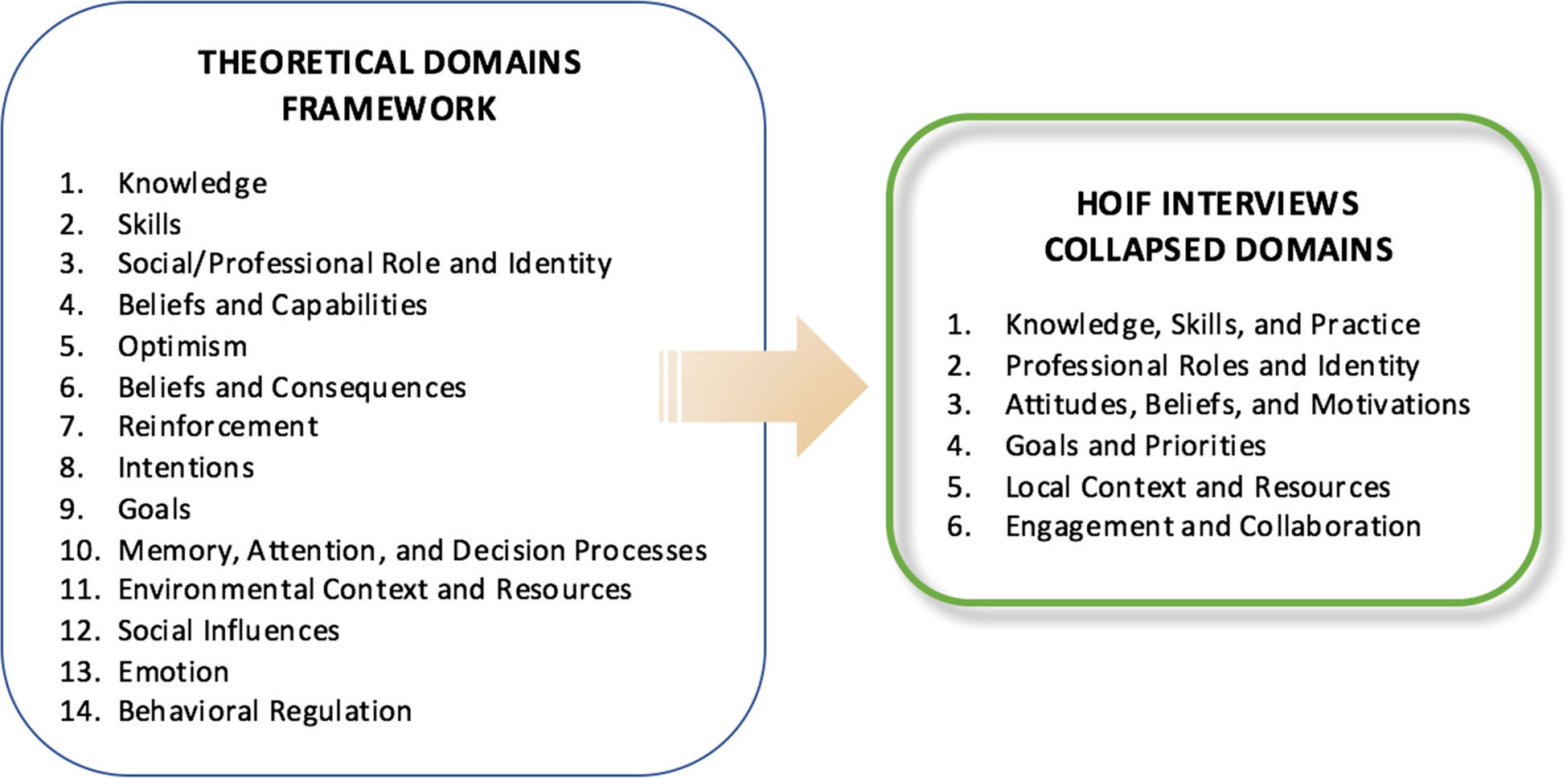
Theoretical domains framework collapsed for project interviews

Physician and nurse interviews revealed significant overlap between responses found in some of the original TDF domains. Hence, after secondary level analyses, the TDF domains were collapsed into 6 clusters to summarize the key findings and identify overarching domains that influence clinical behaviour and CP uptake: 1) knowledge, skills, and practice; 2) professional roles and identity; 3) attitudes, beliefs, and motivations; 4) goals and priorities; 5) local context and resources; and 6) engagement and collaboration (see Fig. 1). Predominant issues that emerged in each cluster are discussed below (see Table 3 for illustrative quotes).

**Table 3:** Clinician perspectives influencing behaviours related to the implementation of a clinical pathway

Domain	Theme	Sample Quote
1. **Knowledge, Skills, and Practice**	Lack of training on concussion Ambiguity around concussion and need for evidence-based info Multiple modalities for knowledge dissemination Practice variation	“I received no formal training … Nobody’s ever talked to me about pediatric concussion. I have received most of it through advanced certification courses … Most knowledge about pediatric concussions is self-directed.” (04RN) “The most difficult thing I find with concussion care at the moment is I don’t necessarily know what the right answer is. Like the research is pretty nebulous as to what we should actually be telling people … there’s a lot of unanswered questions.” (24MD) “Just [use] multiple modalities to reach people. It’s a big team.” (07RN) “There’s just not a lot of consistency in practice right now, so I think having a little more consistency … to guide their orders or their interventions … is always a benefit.” (22RN)
2. **Professional Roles and Identity**	Respecting clinical autonomy Distribution and delineation of roles Managing professional identity and mitigating risks	“Some people might, with pathways, [feel that you are] taking away autonomy from nurses.” (21RN) “Clinical pathways with defined nursing roles can be very successful.” (19RN) “If I’m telling a family that I’m sending a referral to X, Y and Z clinic, they will be contacted within 2–3 days for an assessment in 1–2 weeks, that better happen, because [if it doesn’t], then that destroys your credibility.” (11MD)
3. **Attitudes, Beliefs, and Motivations**	Supportive attitudes and motivations Skepticism, indifference, and potential resistance Attitudes towards research vs. clinical goals	“I think especially at this site, if you say ‘pediatric’, people will jump because it’s not necessarily everybody’s comfort zone here … so a lot of people really jump at the opportunity to get any more pediatric education just to increase their comforts.” (10RN) “We have so many other commitments in terms of clinical work and things like that that sometimes it’s a barrier. It’s just one more thing to do in our days … [we wear] many hats as physicians and many of us are involved in teaching and meetings and committee work and all sorts of things.” (29MD) “I mean it’s still a research project. It’s not mandatory … so that might just be something that takes the back seat when it’s really stressful and busy.” (09RN)
4. **Goals and Priorities**	Enhance patient education and manage expectations Improve coordination of care Streamlining processes	“The thing that parents need most when their kids have concussions is reassurance and some guidelines.” (19RN) “If this pathway tightens up follow-up care after emergency, that would help me immensely. I’ll feel much more confident in making those referrals. So I wish that there was a neater process for that because there’s not many different places I can send them … and the most frustrating is to have a rejection for a referral.” (11MD) “It has to offer [clinicians] something that they’re not getting right now. Like it’s either easier for them or it’s making the decision pathway clearer, or it’s helping them access resources for people … there has to be some benefit to it, or I think it’s going to be a challenge to sell it.” (24MD)
5. **Local Context and Resources**	Practical considerations Site-specific considerations Uniqueness of acute care setting	“From my experience as a manager, you know, the uptake on things isn’t immediate. You have to continue to nurture it and remind and keep going and that’s probably true in most sites if they’re honest about it.” (32RN) “That’s the nature of emergency medicine. We see episodic care. We never find out follow-up … All emergency departments all do the same thing, they only see people once, and they’re more interested in the diagnosis than the follow-up per se.” (15MD)
6. **Engagement and Collaboration**	Stakeholder engagement Other collaborations	“[It is important to] make sure that it’s not being dictated down from the Children’s Hospital, that this is our protocol. Really kind of incorporating and bringing it to the department, rather than just being implemented or forced.” (04RN) “I feel like the most successful pathway is asthma and that’s definitely multi-disciplinary … the strength of the asthma pathway was it not only empowered nursing, but RTs [as well]. So [there were] two groups for keeping this pathway alive and well … [and] multiple champions in different disciplines that provided for its successful outcome, rather than having one or two.” (07RN)

### Cluster 1: knowledge, skills, and practice

Participants unanimously indicated that concussion training is limited in the ED, and that knowledge of evidence-based CPGs is often lacking. Because physicians are responsible for diagnosis and discharge planning, they were more familiar with concussion guidelines and assessment tools than nursing staff. Nurses, however, felt that they could play a larger role in pre-assessment and discharge teaching. They generally saw the study’s CP initiative as an opportunity to learn more about concussion and to clarify the role that nurses can play in concussion care.

Concussion was generally viewed as a controversial topic or one in which research is still evolving. The perceived lack of evidence around concussion, particularly in terms of best practices for concussion management and intervention outcomes, raised some skepticism about the effectiveness of a proposed CP. Staff highlighted the need for the CP to be evidence-based and to demonstrate its clinical utility in terms of both enhancing clinical efficiency and improving patient care and health outcomes.

Participants emphasized that ED staff are already inundated with information; hence, any new information about the CP needs to be targeted, timely, concise, and relevant. Clinicians indicated that a multi-disciplinary and multi-modal approach to knowledge dissemination is vital to reach all staff. Raising awareness of the CP would require working closely with nurse educators and unit managers to distribute information to staff and to implement a reminder system. Staff also identified other avenues for knowledge dissemination outside of the ED that would ultimately be beneficial for improving the coordination of care for concussion patients, such as sharing information with general practitioners (GPs), to whom patients are often referred for follow-up care.

Clinicians acknowledged significant practice variation for concussion care in the ED, and attributed this to factors such as physician preference, clinical practice experience, clinical flow, and lack of practice guidelines. They generally felt that a CP was needed to reduce practice variation and to align practices between nurses and physicians, ultimately to promote more consistent patient care.

### Cluster 2: professional roles and identity

Participants emphasized that respect for clinical autonomy is of vital importance to the success of a CP implementation. Resistance is more likely to occur if clinicians feel that their clinical experience is not being recognized or if the pathway does not result in optimal decision-making for patient care. Clinicians preferred to view the CP as a tool that aids them in their practice rather than as a prescribed set of instructions. They also emphasized the importance of taking into consideration clinical practicality, which means drawing on physicians’ clinical expertise and eliciting their feedback about how best to implement a CP.

Participants emphasized that the CP should reflect a multi-disciplinary, team-oriented approach. They recommended that nurses have a defined role within the CP so that the responsibility for implementation does not fall solely on physicians. Clinicians shared several examples of nurse-initiated CPs that have been used successfully in the ED to reduce physician burden. Moreover, nurse involvement in CPs helps to enhance clinical flow and efficiency.

Participants described how a lack of clarity regarding professional roles, practice variation, lack of coordination of care, and inconsistencies in information provided to patients can all raise the risks of damaging one’s professional reputation and also jeopardizing patient care. They expressed the hope that a CP would help to improve in concussion care in these respects.

### Cluster 3: attitudes, beliefs, and motivations

Participants were generally supportive of a CP for concussion because they agreed it could address a gap in care. Those with less experience working with children were especially supportive of the CP. They were motivated to learn more about concussion in children because they wanted to increase their knowledge and skills and, ultimately, feel more confident working with a pediatric population. The CP was seen as a way to standardize practice, improve continuity of care, and enhance overall quality of care; hence, despite time constraints and other barriers, clinicians generally saw the value of implementing a CP for concussion.

Time constraints and the busy nature of the ED were repeatedly identified as two of the biggest obstacles to CP implementation. ED clinicians may not necessarily be resistant to a CP, but may express scepticism because they are accustomed to working in an environment characterized by constant change and competing priorities. The concept of ‘timely teaching’ was noted, meaning that dissemination of information about the CP and preparation for its rollout should be strategically timed so as to optimize people’s attention to it. Also, clinicians stressed the need to be aware of other initiatives that may be concurrently underway in and around the time of CP rollout, along with the need to determine a strategy, in collaboration with each site, to keep the initiative on clinicians’ radar.

Participants stated that research goals are not necessarily aligned with clinical goals. Some participants felt that research can be impractical, stating how results do not always lead to improvements in clinical care or patient outcomes. Overall, clinicians expressed conflicting views regarding the utility of and support for research, as academically-oriented sites have a larger research infrastructure and are self-described as being a more pro-research environment. Lastly, a distinction was often made between academic sites (e.g., large teaching hospitals) and community-oriented ones (e.g., smaller community health centres).

### Cluster 4: goals and priorities

Patient education on concussion was identified as an important area for improving care. Participants highlighted the need to have one reliable source of information on concussion because patients can easily be overwhelmed with information overload. Practical considerations such as language barriers, educational level, and access to technology were identified at two of the community sites as being particularly relevant for their patient populations. Clinicians felt that a proposed web portal could be a valuable resource for patients and help streamline the process of providing discharge instructions in the ED. However, they emphasized that clinicians should vet the information on the portal, that it should be an improvement compared to other existing resources, and that the information should avoid being vague.

Continuity of care was clearly identified as an area needing improvement, as physicians were frustrated over the lack of adequate follow-up information about their patients. Another concern was not knowing what local resources are available, the criteria for referrals to some specialty clinics, and how long it takes for patients to be seen at a referral clinic. Although ED physicians commonly refer patients to GPs, they are simultaneously concerned with the level of knowledge that GPs have about concussion and how patients will be managed by them. They believe this could ultimately result in unnecessary returns to the ED, increased levels of stress in patients, or inappropriate referrals for diagnostic imaging. Overall, participants viewed the CP as an opportunity to provide clarity on local referral options.

Participants emphasized that the CP needs to be ‘value added’, meaning that, among other things, it should help to streamline concussion care in the ED. For example, it ought to be evidence-based, clear, concise, efficient, easy to locate, avoid duplication of charting, and easily integrated into current practice. It should offer something novel to improve care, otherwise staff would resist adopting it. Some participants suggested that the CP could be particularly helpful in providing clarity as to how to stratify patient management of concussion based on age and severity of the concussion.

### Cluster 5: local context and resources

Clinicians highlighted several practical considerations for CP implementation, including the timing of the CP rollout, personnel shortages, use of site champions, data ownership, operational approvals, training requirements, accessibility of forms, documentation and charting, and usage of reminder or alert systems. These practical considerations reflected the concrete and everyday needs, operations, or limitations of the ED for implementing a CP.

Factors that are unique to the ED, and which could affect CP implementation, include clinical flow, the nature of episodic care, structural constraints, and the hectic pace and constant change in the ED. A key point highlighted by clinicians is that the ED offers episodic care rather than focusing on follow-up care. This feature has implications for the uptake of the CP, as clinicians may feel that the CP is not relevant to the ED context if the CP focuses on patient education and follow-up care.

### Cluster 6: engagement and collaboration

Stakeholder engagement was identified as critical to the success of CP implementation. Key staff, such as unit managers, nurse educators, physicians, and site chiefs, need to be consulted and given the opportunity to provide feedback on the CP and the implementation strategy for them to endorse the initiative. Staff are more likely to support the project and ensure the uptake of the CP if they are well-informed about it. The ideal site champions were felt to be those who have a keen interest in pediatrics and are more involved in clinical rather than administrative work. Clinicians stressed the importance of clearly articulating the goals of the CP to garner more support for its implementation.

Participants identified opportunities for other collaborations that could be beneficial for the CP initiative. For example, any concussion handouts developed for patients should align with existing resources provided through provincial health agencies. Additionally, collaboration with referral clinics could increase uptake of the CP by improving coordination of care, which was identified as being problematic in pediatric concussion.

## Discussion

While this study was not intended to systematically identify intervention functions or behaviour change techniques as some studies have done according to the Behaviour Change Wheel (BCW) framework [39–42], we found that by adopting the TDF as an analytical framework, we were able to successfully identify the facilitators and barriers that would impact the implementation of a CP for pediatric concussion in emergency departments. The 6 clusters of TDF domains identified in the interviews each reflect 2–4 predominant concerns that can be condensed into six overarching themes regarding clinicians’ views on the implementation of a CP. We believe that these themes are essential to consider and address in any CP implementation project in settings characterized by shifting priorities, multidisciplinary teams, and evident practice variation.

### Standardization in the midst of evolving research

A predominant concern, particularly among physicians, is the belief that research-derived evidence about concussion is lacking or still in its infancy. Concussion was deemed to be a controversial topic that does not lend itself well to a CP because the research continues to evolve and clinicians are unsure of which interventions will be most effective. On one hand, clinicians want to support further research to build evidence for best practices and effective interventions. On the other hand, they are sceptical of supporting efforts to standardize clinical practices when they believe that the research is still evolving or that debates persist over the evidence. These conflicting views about the purpose and utility of a CP were also found in O’Hara and colleagues’ study of pediatric hospitalists and emergency medicine physicians’ perspectives of CPs [43].

Thus, any plan for CP implementation must highlight how the CP will address gaps in the evidence, as clinicians are more likely to adopt a CP if they see that medical and research experts in the field have carefully reviewed the research evidence and considered its clinical application, a view echoed in Craig and colleagues’ implementation study of behaviour change techniques in the delivery of care to stroke patients in EDs [44]. Clinicians in our study also emphasized the importance of bridging the research-to-practice gap. Thus, implementation of a CP is more likely to be successful if practitioners are shown how, based on evidence-based research, a CP can improve care, patient health outcomes, and clinical efficiency.

### Clarifying and communicating goals

A striking finding was the evident lack of clarity around what constitutes a CP. Several physicians asked for clarification about how the study investigators defined a CP. They also insisted that the goals of a CP versus those of a research study should be kept distinct, explicitly defined, and clearly communicated to clinicians at the participating sites. This may help to explain some clinicians’ resistance to a CP, as other studies have shown that potential barriers to implementation include differing views on the applicability of CPs or lack of awareness or communication regarding guidelines and their efficacy [43, 45, 46]. Thus, the goals of the CP must be clearly communicated to clinicians in terms of patient assessment and management of care, clinical flow and organization, and availability of resources. Study investigators can also emphasize how a CP will offer a tool to clarify clinical roles, align practices, and support staff in providing consistent and quality care.

The interviews also identified a need to better understand the personal and professional goals of clinicians themselves, to provide insight into the levels of engagement and interest that they might bring to adopting the CP. For example, very few nurses interviewed had significant experience working with children, and even those who practiced at a designated pediatric site were relatively new to the setting. However, nurses expressed a strong desire to gain confidence and skills in working with children. Thus, the CP initiative presents a learning opportunity. Feedback from participants inspired our research team to assemble a list of resources on concussion care to share with clinical staff while also generating discussion about the possibility of designing a multi-purpose web portal for both patient use and clinician access to training modules.

### Knowledge dissemination and aligning information

Knowledge dissemination and the need to consolidate existing concussion information were frequent interview topics. Knowledge dissemination here refers to both patient education and informing practitioners about the CP. Regarding the former, two key points arose: 1) taking into account the unique characteristics of the patient population at each of the clinical sites in preparing educational resources; and 2) standardizing information so that all patients receive the same reliable information on concussion.

Interviews revealed that the patient population at two of the community health centres are comprised of a large percentage of individuals who are non-English speaking immigrants. This language barrier has implications for discharge teaching and needs to be considered in preparing informational resources, such as patient leaflets. While English is a barrier for some parents, their children are usually functional in English and often act as interpreters for their parents. Hence, children will likely be responsible for accessing health information on their own behalf, and will need to navigate the available concussion information on their own accord. Lastly, online concussion information may be effective for families for whom English is a second language. In the context of a highly stressful ED visit for a concussion diagnosis, parents are often not able to fully grasp the information provided to them upon discharge. This stressful context, in conjunction with language barriers, makes the verbal communication of concussion information during discharge teaching largely ineffectual for immigrant patients and families.

Clinicians welcomed the idea of having a single, reliable, evidence-based source of information to provide patients and parents. A common suggestion was to ensure that any information (e.g., printed brochures, online web portal) developed for patients be aligned with other key provincial health resources. Overall, the standardization of concussion information is an aspect of the proposed CP that was especially appealing to clinicians, because it can help streamline discharge teaching.

The dissemination of information about the CP to practitioners is one of the more challenging aspects of implementation. Uptake of the CP requires, first and foremost, adequate exposure to information about the pathway, an understanding of its potential benefits, and personal motivation to modify clinical behaviour. These findings are consistent with other studies showing that a lack of awareness of guidelines or perceived lack of evidence for measurable change represent a barrier to CP implementation [45, 47, 48]. Other researchers have highlighted the importance of developing educational material for clinical personnel [43], designing innovative strategies to optimize clinician education in high stress and fast-paced settings [41], and focusing on training and education as key intervention functions for enabling behaviour change [39, 40].

Clinicians strongly advocated a knowledge dissemination strategy that uses multiple modalities. Practical suggestions included targeting different shifts, using site champions to disseminate information, giving presentations to both physician and nurse groups, sending electronic notices, posting bulletins in newsletters, putting up posters in the ED, doing site visits prior to and after the CP launch, attending in-service sessions, and devising other alert systems using existing resources at each site.

### A team-oriented approach

Physician and nurses alike emphasized the value of taking a multi-disciplinary, team-oriented approach to CP implementation. Successful CP implementation was judged largely contingent on having staff in various roles, from physicians to unit clerks, be aware of the CP implementation and to act as reminders for colleagues. This finding aligns with other studies that endorsed a “health care provider leadership model” to facilitate communication, provide peer support, receive feedback, and improve CP uptake [45].

A team-oriented approach also reduces the burden of responsibility for CP implementation on specific individuals. Physicians expressed concerned that the introduction of a CP would add to their workload, while nurses emphasized their wish for a more active role in both concussion care and the implementation of CPs. Nurses embrace their role as patient educators and want to ensure that patients and families have adequate health information upon discharge. Hence, they see an opportunity within a CP to assume a more defined role in discharge teaching. Additionally, many nurses expressed interest in taking a more active role in patient pre-assessment and to learn more about concussion. They noted that initiatives to translate evidence-based research into practice are often oriented primarily to physicians, while affording nurses only cursory information and limited involvement.

### Site engagement

Clinicians discussed how site engagement is critical to the success of CP implementation, so that they are able to provide critical feedback on the feasibility and clinical utility of the CP. Collaboration ensures that sites will be more motivated to achieve the goals of the CP implementation team. This finding aligns with other studies in emphasizing the importance of stakeholder engagement, including the opportunity it affords to identify early adopters or “change champions”, engage senior leadership, establish consensus, and share local knowledge [41–44]. Site engagement also ensures that pertinent information about site-specific considerations are shared with the research team, such as competing priorities in the ED, changes in protocols, operational approvals, personnel changes, and local resources.

Several clinicians spoke candidly about issues such as “research fatigue” or how staff do not take well to external research studies that they feel have been imposed on them, especially at community health sites, which may reluctant to adopt initiatives introduced by larger academic hospital sites. This apparent tension between the two types of clinical facilities speaks even more to the need for site engagement, to ensure that each individual site is given the opportunity to share its unique needs, environmental context, and available resources. A common view held by nurses was that they have not been actively involved in consultation and planning on previous CP initiatives, despite being critical to their success. Nurses play a key role in disseminating information to staff, reminding physicians about the new CP, and facilitating implementation through initial patient screening and discharge teaching.

### Streamlining clinical processes

Clinicians stated emphatically that a CP would not be well-received if it did not help to streamline clinical processes. Given the hectic ED environment, clinicians look for how new initiatives make a “value added” contribution to routine clinical practice. Physicians are concerned that a CP will be cumbersome or impede clinical efficiency. They wish to avoid, for instance, the duplication of charting, lengthy and unnecessary patient assessment and history taking, and prolonged discharge teaching. “Value added,” in this context, refers both to improving patient care through evidence-based practices and to facilitating clinic flow by reducing the amount of time clinicians need to spend on administrative tasks. This finding suggests that successful CP implementation depends in part on showing how a CP supports and enhances existing practices, rather than involving more daunting or intrusive changes.

### Reflection on using the TDF

The results of the interviews described here represent the initial steps needed to inform the development and implementation of a CP that aims to improve pediatric concussion care. Feedback from clinicians on the feasibility and practical considerations of CP implementation have proven highly valuable in guiding the next phase of the larger parent study. Given how time-consuming and complex implementation studies can be [36], this initial research helped to identify clinical behaviors, attitudes, and other environmental factors that must be considered to ensure the successful uptake of the CP in busy ED settings. The application of the TDF framework allowed us to develop a comprehensive, semi-structured interview guide, and also served as a useful tool to organize and structure the analytical framework to identify emerging themes and overlapping areas of concern identified by participants. As other studies have noted, the TDF allows for a focused and efficient means of data analysis [28] and is particularly useful for identifying and grouping general sets of beliefs into comprehensive domains that are based on validated behavior change theories. Most importantly, the TDF serves as an effective link between theory-based investigation and intervention in a clinical setting [22].

While the TDF provided a comprehensive framework for the interview guide, we found significant overlap between some of the domains based on participants’ responses, leading to some redundancy in the interviews. Other investigators have remarked on related limitations [49, 50]. When questions regarding beliefs, intentions, goals, and emotions are distributed across different domains, participants’ responses end up being heavily weighted to those areas by sheer virtue of the frequency with which they appear in the TDF. Hence, in our analysis, TDF domains were clustered to reduce redundancy.

Another limitation is that the TDF is perhaps too prescriptive, and may preclude the analysis of qualitative data in an inductive manner that allows themes to emerge from participants’ own concerns and insights. A salient finding from the interviews, for example, was the recurring question of what actually constitutes a CP, what are its goals, and why are clinicians asked to change their clinical behaviour when they do not feel a need to do so. These responses helped the study team re-examine its a priori assumption that clinicians have a clear idea of what a CP entails and that the adoption of a CP, with related changes in clinical behaviour, was needed to improve care for concussion. Instead, participants’ responses suggested that such an approach could be viewed as threatening or dismissive by experienced clinicians, who suggested that the CP implementation ought to be reframed as the presentation of tools to *support practice* rather than to *change behaviour*.

Ultimately, the TDF provides a useful starting point as a comprehensive, evidence-based, and theory-driven framework for developing a sound guide to structure open-ended qualitative interviews regarding the potential implementation of new clinical practices. Then, when used flexibly as an analytical tool to guide data analysis, the TDF can be employed in conjunction with a more inductive thematic analysis approach that allows for themes to emerge from participants’ responses themselves, rather than forcing them to map onto predetermined domains. Using this flexible approach, novel findings may arise, as was the case in our study, providing important insights into clinicians’ views on the goals and challenges of implementing a CP.

## Conclusion

Application of a comprehensive, evidence-based, and theory-driven framework in conjunction with an inductive thematic analysis approach enabled six themes to emerge as to how to best implement a concussion clinical pathway. These overarching themes must be addressed to successfully implement a CP for pediatric concussion.

## Data Availability

The datasets used and/or analysed during the current study are available from the corresponding author on reasonable request.

## Abbreviations

AHS: Alberta Health Services
CP: clinical pathway
CPG: clinical practice guidelines
ED: emergency department
GP: general practitioner
HOIF: Health Outcomes Improvement Fund
MNCY: Maternal Newborn Child and Youth
SCN: Strategic Clinical Network
TDF: Theoretical Domains Framework
